# Chemical Compounds Related to the Predation Risk Posed by Malacophagous Ground Beetles Alter Self-Maintenance Behavior of Naive Slugs (*Deroceras reticulatum*)

**DOI:** 10.1371/journal.pone.0079361

**Published:** 2013-11-14

**Authors:** Piotr Bursztyka, Dominique Saffray, Céline Lafont-Lecuelle, Antoine Brin, Patrick Pageat

**Affiliations:** 1 Department Agronomy-Aquaculture, Research Institute in Semiochemistry and Applied Ethology, Saint-Saturnin-lès-Apt, Vaucluse, France; 2 Biodiversité des Systèmes Agricoles et Naturels UMR 1201 Dynafor, Engineering School of Purpan, Toulouse, Haute-Garonne, France; College of Charleston, United States of America

## Abstract

Evidence that terrestrial gastropods are able to detect chemical cues from their predators is obvious yet scarce, despite the scientific relevance of the topic to enhancing our knowledge in this area. This study examines the influence of cuticular extracts from predacious ground beetles (*Carabus auratus*, *Carabus hispanus*, *Carabus nemoralis* and *Carabus coriaceus*), and a neutral insect species (*Musca domestica*) on the shelter-seeking behavior of naive slugs (*Deroceras reticulatum*). Slugs, known to have a negative phototactic response, were exposed to light, prompting them to make a choice between either a shelter treated with a cuticular extract or a control shelter treated with pure ethyl alcohol. Their behavioral responses were recorded for one hour in order to determine their first shelter choice, their final position, and to compare the percentage of time spent in the control shelters with the time spent in the treated shelters.The test proved to be very effective: slugs spent most of the experiment in a shelter. They spent significantly more time in the control shelter than in the shelter treated with either *C. nemoralis* (Z = 2.43; p = 0.0151; Wilcoxon matched-pairs signed-ranks test) or *C. coriaceus* cuticular extracts (Z = 3.31; p<0.01; Wilcoxon matched-pairs signed-ranks test), with a seemingly stronger avoidance effect when presented with *C. coriaceus* extracts. The other cuticular extracts had no significant effect on any of the behavioral items measured. Although it cannot be entirely excluded that the differences observed, are partly due to the intrinsic properties of the vehicle employed to build the cuticular extracts, the results suggest that slugs can innately discriminate amongst different potential predators and adjust their behavioral response according to the relevance of the threat conveyed by their predator’s chemical cues.

## Introduction

Among the most essential needs for organisms, the ability to detect and avoid predators is paramount [1], [2] because the consequences of predation are often irretrievable and dramatic for the lifespan – and hence the fitness – of prey species [3]. Predation is considered to be a major selection force that drives organism evolution [4]–[6] and intervenes in every phase of life by shaping morphology, behavior, ecology and life history traits [2], [4], [7].

It is to the benefit of prey to be able to assess, at any time, the actual threats posed by predation in order to adequately adjust activity rather than expend large amounts of energy in an attempt to escape a direct attack from a predator. Prey species may use many sensory modalities to track down any cues that betray the presence of a predator, but olfaction appears particularly suitable to fulfilling this task. Indeed, one of the main advantages of chemical cues is that they can be perceived from a distance, with no contact of any kind with the predator.

There is extensive literature on chemically mediated antipredator behaviors within aquatic organisms [8], [9]. Because aquatic environment are frequently turbid, water-borne chemical cues are often the only reliable cue available to prey [1]. These chemicals can be dispersed in large volumes of water, even in very low amounts, increasing their likelihood of being perceived by target organisms [10]. Thus, if we consider that chemical senses are the oldest and the most ubiquitous form of sensory perception, it is not surprising that predation risk assessment through olfactory means is so common in aquatic environments [1], [11], [12].

Predation risk assessment through olfaction among organisms living in terrestrial ecosystems appears to have been investigated to a lesser extent [1]. However, most if not all terrestrial animals draw on chemical cues for a wide range of essential interactions with their environment, including managing the threat of predation. This is especially true among terrestrial invertebrates whose alternative vigilance senses, such as sight or mechanical stimuli, are usually less accurate than their counterparts found in vertebrates, or simply non-existent. Storm and Lima (2008) [13] pointed out the lack of investigation on the recognition of predator chemical compounds among insects despite the prominent importance of chemical communication within this class as evidenced by the great diversity of their chemoreceptors [14]–[17]. The same observation can be made regarding terrestrial gastropods.

While chemically-induced antipredator behavioral responses have been extensively documented within aquatic gastropods [1], [18]–[22], very few have focused on how related terrestrial species perceive their foes. It is well-known that, like aquatic gastropods, terrestrial gastropods rely mostly on olfaction [23]–[29]. Terrestrial gastropods have no acoustic sense and their eyes are largely inefficient as they appear to only be capable of distinguishing light and dark areas [27], [29]–[32]. Consequently, critical behavioral decisions appeal to chemical cues gathered from the environment. Predation risk assessment is likely to be one of the most significant of these.

Terrestrial gastropods represent a regular food source for many different animals [33], particularly for ground beetles [34]. Only two studies have addressed the question of behavioral responses of terrestrial gastropods in the presence of chemical cues from two species of ground beetles. Studies show that the snail *Theba pisana* (Müller, 1774) remains stationary longer and climbs fastest in the presence of feces from *Carabus carabus* (Heller, 1993) fed with snails than in presence of various controls [35]. In another study, *Deroceras reticulatum* (Müller, 1774), a slug species, avoided paper that had been exposed to *Pterostichus melanarius* (Illiger, 1798), a common generalist predator in European fields, indicating the putative presence of a repulsive kairomone [36].

Ground beetles have been extensively studied for their ability to control populations of *D. reticulatum* [37]–[43] because of the detrimental impact of the slug species on several agricultural crops [28], [44]. The beetles may also provide an ecological alternative to the usual chemical means of control, which present a number drawbacks [45]–[49]. Most of these studies focused on carabid beetles commonly found in fields, with the most representative species belonging to the subfamilies Nebrinae, Harpalinae, Pterostichinae, and Zabrinae [38], [50]–[55]. Despite numerous direct and indirect observations attesting to the consumption of slugs by these species (see Symondson (2004) [34]), it appears that their predatory capabilities are largely confined to small slugs (i.e. young slugs) [39], [50], [56]–[58]. Surprisingly, few studies have examined the ground beetles from the subfamily Carabinae. However, the largest ground beetles belong to this subfamily and some evidence indicates that they possess superior skills in managing the main defense mechanisms displayed by slugs, such as heavy mucus exudation and autotomy [58]–[60]. This suggests that these predators may exert stronger selection pressure on slugs than more generalist feeders, like *Pterostichus spp.*

Taking into account these observations, the aim of this study was to evaluate whether chemical cues from *Carabus auratus* Linnaeus, 1761, *Carabus hispanus* Fabricius, 1787, *Carabus nemoralis* Müller, 1764, *Carabus coriaceus* Linnaeus, 1758, all four belonging to the Carabinae subfamily, could affect the behavior of slugs. Predator chemical signals can affect prey behavior in various ways. For instance, prey can adjust its movements (mobile or static, speed variation) [8], [13], [61]–[67], sheltering [68] or shoaling [69], [70]. This study assesses the perception of chemical signals left by these grounds beetles in *D. reticulatum* by examining behavioral alterations in the search for shelter. Devoid of the protective shells of snails, slugs generally find protection under shelters, shielding them from the unfavorable conditions that generally prevail during the day due to heat, sunlight, or even drafts. Slugs are thus mainly active at night [28], [71]–[73], whilst they principally seek shelter during the daytime [27], [28], [74]–[76]. Correlatively, it has been shown that slugs prefer dark areas to light ones [30], [74]. Thus, an experimental choice test was conducted first, prompting the slugs to choose between two refuges under the influence of light. Secondly, cuticular extracts of ground beetles were deposited in one of the two shelters in order to assess whether the presence of these cues would drive the slugs to preferentially choose the control shelter.

## Materials and Methods

### Biological Material

#### Species of interest for the study

The slug from the species *Deroceras reticulatum* (Müller 1774) is a well known worldwide pest which hence is neither endangered nor protected. Adults were caught in fields and private gardens not subjected to regulatory protection in the vicinity of Apt (84400 Vaucluse, France) during spring 2011, with the agreements of the owners.

Carabid beetles from the species *Carabus auratus*, *Carabus nemoralis* and *Carabus hispanus* were captured in spring, and *Carabus coriaceus* were trapped in fall, in private gardens with the agreement of the owners. These all four ground beetles are neither endangered nor under law protection in France.

The snail species *Xeropicta derbentina* (Krynicki, 1836), which served to feed the ground beetles, is an alien species in Provence, France. It is thus not protected by law and was captured on the research institute’s ground.

The house flies *Musca domestica* is a common species of the Diptera order found worldwide which is therefore not endangered nor subjected to any form of regulation. Adult flies were caught on the research institute property where the experiments were done.

### Rearing of the Species

The slugs from the species *Deroceras reticulatum* (Müller 1774) employed for the study were raised from eggs laid by the collected adults. The slugs were housed in plastic boxes (L: 20 cm/l: 10,5 cm/h: 8 cm) lined with wet paper at 17°C +/−1°C and 80% RH +/−5% and were submitted to an L:D cycle of 11 hours and 13 hours respectively (lights on from 09∶00–20∶00). Twice a week, the boxes were cleaned and the slugs were fed lettuce and a supplement of commercial dry rabbit food (Coqtel). Slugs were reared in laboratory and were thus never exposed to their predators, notably carabid beetles, and can be considered as “naive” regarding such experience. Experiments were carried out with slugs in the intermediate life stage, as determined by body weight (ranging from 0.1 to 0.2 g) [36].

Ground beetles individuals from the same species were then collectively housed in a plastic box (l: 55 cm/w: 35/h: 14.5 cm) lined with 4 cm of moist loam covered by moss and placed at 20°C +/−2°C with an L:D cycle of 8 hours and 16 hours respectively (lights on from 10∶00–18∶00). They were fed daily with the land snail *Xeropicta derbentina* (Krynicki, 1836).

The house flies *Musca domestica* (Linnaeus, 1758) were reared in laboratory. The imagos were maintained in a screened enclosure in a room at 22°C +/−3°C, with 50% RH +/−10%, and were subjected to an L:D cycle of 14 hours and 10 hours respectively and fed a 5 g mix of 50% castor sugar and 50% milk powder (Modern Veterinary Therapeutics formula for kittens and puppies, 25% protein and 24% fat), which was changed weekly. Hydration was provided by a water-soaked paper towel. Egg clutches were obtained on 5 to 10 g of chicken manure, first dried and then rehydrated to 70% RH. Eggs and then larvae were housed in the same previous substratum at 30°C +/−2°C and 50% RH +/−10% in a terrarium with a L:D cycle of 14 hours and 10 hours respectively. Only imaginal life stage organisms obtained from this laboratory breeding were used in the experiment.

### Production of the Stimuli

During their captivity, some of the ground beetles died, whether due to age (unknown at time of their capture) or any other reasons unknown. Carabid beetles were checked twice a day (once in the morning and once in the late afternoon) to ensure vitality. Those found freshly dead due to an apparently natural cause (i.e. other than cannibalism, accident or infection) were withdrawn from the breeding box and placed in a plastic beaker filled with pure ethyl alcohol (99.8%) depending on the species size to which the carabid beetle belonged: 15 ml for each *C. auratus*, *C. nemoralis* or *C. hispanus,* but 20 ml for each *C. coriaceus* as it is a larger species. These “stock solutions” were stored in a refrigerator at 4°C and gently shaken once a week during a variable time depending on the availability of the ground beetle species: about 6 months for *C. hispanus*, 4 months for *C. auratus* and 3 months for *C. nemoralis*, *C. coriaceus* and flies. Despite this seemingly long storage time, it has been shown that similar preparations of body extracts from cockroaches kept their property for at least one month [77], [78]. We collected 2 ml of each stock solution in separate glass vials, just before the beginning of the trials. These working solutions were stored at 4°C and served throughout the trials, *i.e.* about two weeks. The study originally included carabid beetles from both sexes. However, due to breeding contingency and the period in which the present study was performed, only females were available for some of the carabid species in the laboratory’s possession. Since Armsworth *et al.* (2005) [36] observed that slugs display no behavioral differences to treatments from male or female *Pterostichus melanarius* (Illiger, 1798), the trials were carried out with extracts from one ground beetle of female sex for each species – the only sex available for all ground beetle species at the time – in order to create a homogenous group of cuticular extracts, bearing in mind that sex would not have an incidence on the slugs’ behavioral response. In addition, a cuticular extract was produced from *Musca domestica* because it is a taxonomically distant insect species and has a very different diet from ground beetles. A mixture of male and female flies (for a total of 15 individuals) were killed by frost (−18°C for one day) and then transferred into 15 ml of pure ethyl alcohol.

### Behavioral Choice Experiments

#### Validation of the experimental design

**Figure 1:**
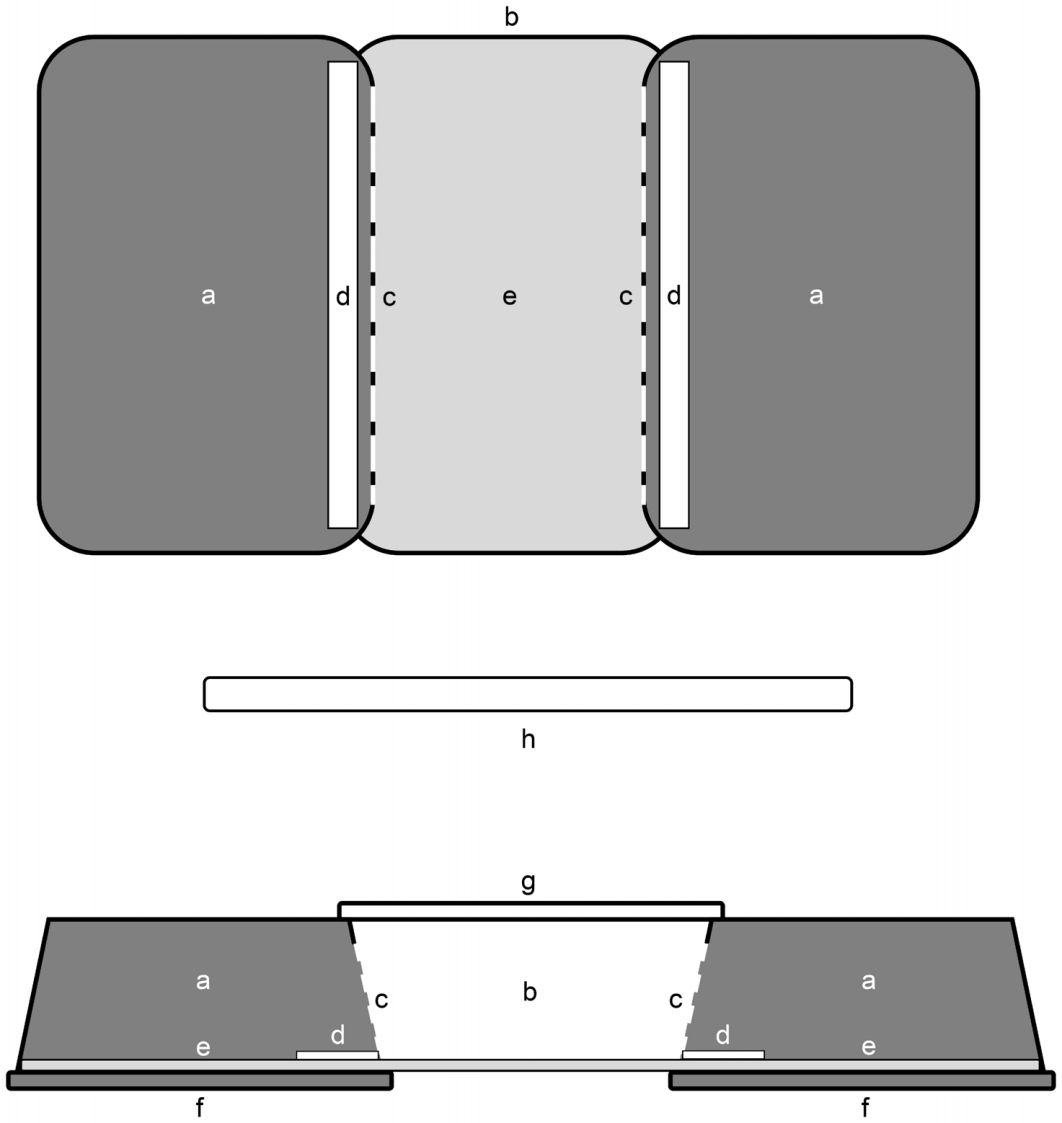
Top and side view of an experimental arena. *a:* dark shelter (3.8+/−0.4 lumens); *b:* lit area (221+/−5 lumens); *c:* shelter entrance; *d:* strip of Whatman paper with 100 µl of either a tested cuticular extract or control; *e:* undersheet moistened with 10 ml of tap water; *f:* dark lid; *g:* translucent lid; *h:* daylight tube (6500°K) 80 cm above the experimental arenas.

The study involved a choice experiment designed to be convenient, easy to set up and efficient. Slugs were led to choose between two shelters, fulfilling the previous criteria while providing a very conspicuous behavior to evaluate the effect of the cuticular extracts on such a choice. The trial was carried out using handmade devices, hereafter referred to as “arenas” (fig. 1), made from sterilized boxes of transparent polystyrene typically used for growing plants (Dutscher, Ref.: 017001). Each arena was composed of three plastic boxes. The finished arena measured 27.5×14.5×4.5 cm. The three boxes were fitted together lengthwise. The two side boxes (shelters) were placed upside down with the lid of the middle box (lit area) resting on the bottom of the side boxes, creating a single arena with three compartments. The side trays were completely darkened by coating their external walls with black tape to fulfill the role of shelters, whereas the central one was left transparent to allow the light to pass through. An opening measuring 1.5×8 cm was cut on each side connecting the central part with the side boxes to allow slugs to access either of the two shelters. The bottoms of arenas were lined with an undersheet (Hartmann Molinea® Plus) moistened with tap water to provide a convenient and comfortable surface for crawling while maintaining a high humidity (about 99% RH) in each area of the arena. Ten arenas were built in order to perform ten replicates at the same time. The experiment took place in a still air room at a temperature of 15.5°C +/−1.5°C and an ambient relative humidity of 72% +/−5%, close to the optimum conditions for slug activity [73], [79]. One slug of medium size was placed in the middle of the lit area of each arena under a red light rather than directly under a white light so as to minimize the burst of activity induced by white light at the beginning of a trial. Slugs were placed so that the body was parallel to the shelter entrances. Once each slug had been placed, a white light (6500°K, 220+/−5 lumens) was turned on and left lit for the duration of a replica (*i.e.* one hour) in order to create a burst of activity that spurred the slugs to seek shelter. Slug movement was recorded for one hour with two digital cameras (JVC HD Everio GZ-HM446) from the moment the slugs were placed under the red light.

### Behavioral Choice Experiments in the Presence of Different Treatments

As slugs spent most of the time sheltered during the pretest (see results), tests were conducted to determine the effect of five cuticular extracts on slug shelter choice. The same device as described above was used with the following changes: six of the arenas were employed simultaneously. Slugs thus had to choose between one of the 6 experimental shelters (5 cuticular extracts or a blank) and a control shelter (pure ethyl alcohol at 99.8% used to produce the cuticular extracts). Treatments (i.e. cuticular extracts or ethyl alcohol) were applied at the rate of 100 µl using a micropipette, on small pieces of Whatman paper (grade 1) measuring 1.5×9 cm. The blank received no treatment (piece of Whatman paper alone). All the pieces of Whatman paper were left at room temperature for 12 minutes in order to allow the excess ethyl alcohol to evaporate. Based on findings by Chase (1982) [80], snails appear unable to detect ethanol through olfaction. It is thus reasonable to assume that slugs cannot detect it, but the high residual quantity of ethyl alcohol on paper strips may interact with the foot of the slugs when crawled upon and could therefore alter behavior. Once the alcohol had evaporated, a treated paper strip was placed in one of the two shelters in each arena using clean forceps, while the remaining shelter received a control paper strip. Each treatment was tested 24 times for one hour each time, and a new slug was used for each replicate. The location of the treatment and control were reversed for each new test and treatments were rotated from arena to arena in order to avoid both shelter and arena position bias. Trials were made with the same conditions of temperature and humidity as the pretest, the slugs used were of the same size, and the same precautions were taken at the beginning of each replicate. The head orientation of the slugs was reversed for each new replicate to avoid orientation bias. Once each slug had been placed, a white light (6500 K, 220+/−5 lumens) was turned on and slug movement was recorded for one hour using the same digital cameras as during the pretest. Two sets of replicates were performed between 9∶00 and 12∶00 every morning until a total of 24 replicates was reached for each of the 6 conditions. Arenas were thoroughly washed and dried in open air for 24 hours between each set of tests.

### Statistical Analysis

Video recordings were used to determine several parameters based on slug movement. Only replicates where slugs went into at least one of the shelters and in which slug movement in the lit arena was clearly visible for the duration of the experiment were retained. Replicates for which those conditions were not fulfilled were retested with the corresponding treatment using a new slug. Each treatment was thus tested 24 times against a control, without any loss of data.

To assess the relevance of the choice test design, the percentage of time spent in the sheltered area versus the time spent in the lit area was calculated as follows:
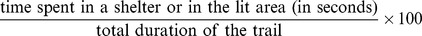
and the proportion of time spent in each shelter was calculated as follows:



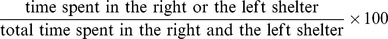



These two behavioral parameters showed no significant deviation from a normal distribution (Wilk–Shapiro normality statistic), so a paired t test was used. The first shelter chosen by each slug and its position at the end of the trial (right/left shelter or treated/control shelter) were noted and significant differences between shelter preferences were evaluated using the McNemar test.

The same criteria as described for the pretest validation were used regarding the assessment of the treatment’s effect on the slugs’ choice of shelter. But as the normality was not ascertained using the percentage data, a Wilcoxon matched-pairs signed-ranks test was used. Shelter preferences were also evaluated using the McNemar test. In addition, a one-way ANOVA was used to compare the different treatments. Data were transformed according to their nature to meet the conditions for an ANOVA. Data was recorded for access latency to the first refuge, total time spent sheltered, time spent in the treated shelter, and time spent in the control shelter. All these durations, expressed in seconds, were log-transformed [81]. The numbers of outings and the numbers of shelter permutations were noted and both of these were square-root transformed to improve normality. Lastly, ratio data was compared after transformation according to the arcsine of the square root [82]: the total time spent sheltered over total trial duration, the time spent in the treated shelter over total trial duration, the time spent in the control shelter over total trial duration, the time spent in the treated shelter over total time spent sheltered, and the time spent in the control shelter over total time spent sheltered. Data analyses were carried out with Statistica 10.0 software and the significance threshold was classically fixed at 5%.

## Results

### Validation of the Experimental Design

**Figure 2:**
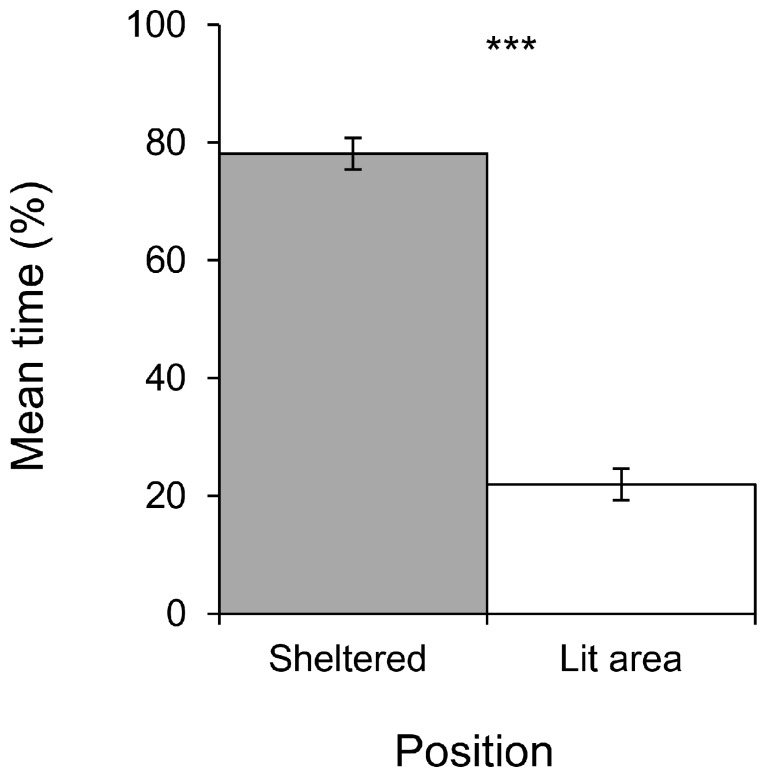
Comparison of the mean percentage of time spent by slugs sheltered with the mean time spent in the lit area. Data employed for the validation step of the design (n = 10, ***P<0,001, paired t test). Bars = ±1 SE, n = 10.

**Figure 3:**
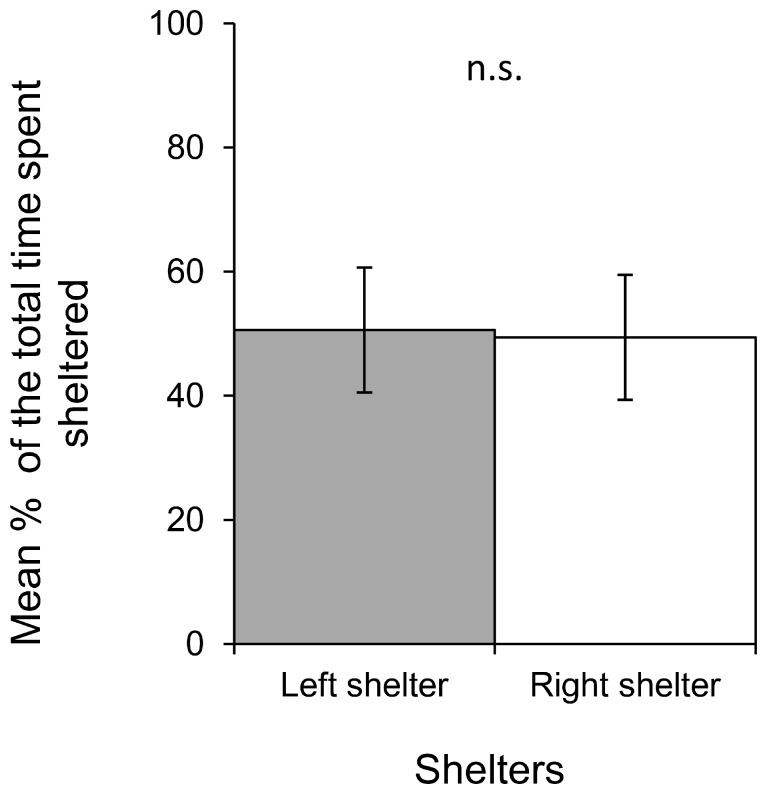
Comparison of the mean percentage of time spent by slugs in either the left shelter or the right shelter. Data employed for the validation step of the design (n.s. not significant, paired t test). Bars = ±1 SE, n = 10.

**Figure 4:**
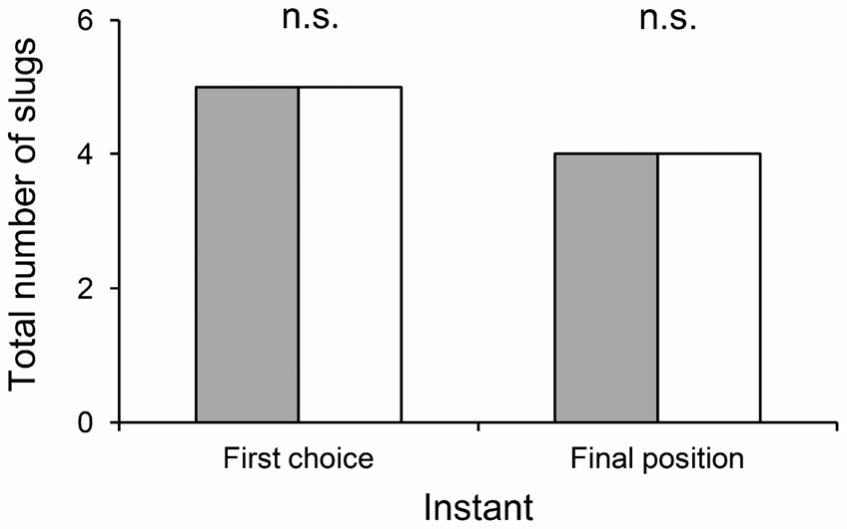
Total number of slugs having chosen either the left or the right shelter as their first choice and at the end of the experiment (n = 10, n.s. not significant, Mc Nemar’s test). Solid bars = left shelter; empty bars = right shelter Data collected from ten replicates.

Slugs spent significantly more time sheltered throughout the duration of a replicate than in the lit area (P<0.001), with an average of 78% spent in a shelter compared to 22% spent in the lit area (figure 2). The slugs showed no preference for either the left or the right shelter (p = 0,954) (figure 3). In addition, slugs did not show any preference for one of the two shelters, neither for their first choice of location or their final location at the end of the experiment (p = 1) as shown in figure 4.

### Shelter Choice Test Results

**Figure 5:**
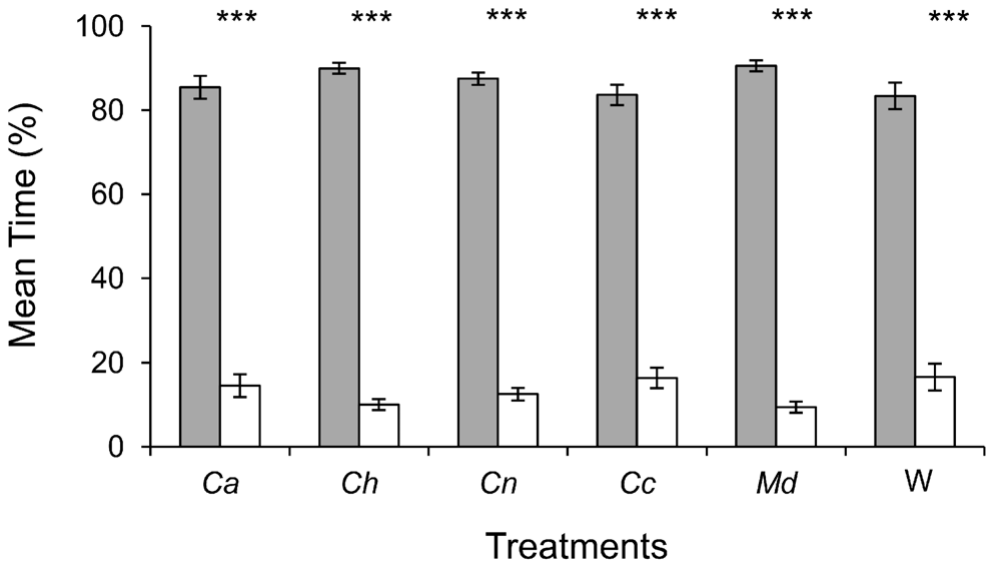
Comparison of the mean percentage of time spent sheltered (expressed as the mean time spent sheltered over the total trial duration) with the mean percentage of time spent in the lit area (expressed as the mean time spent in the lit area over the total trial duration), according to each treatment. Treatment applied in the treated shelter (dark boxes) for each test arena is indicated below each histogram pair: *Ca* (*Carabus auratus*), *Ch* (*Carabus hispanus*), *Cn* (*Carabus nemoralis*), *Cc* (*Carabus coriaceus*), *Md* (*Musca domestica*), W (strip of Whatman paper alone). Empty bars are the control (pure ethanol). Asterisks indicate significant differences: ***P<0.001. Bars = ±1 SE, n = 24 for each treatment.

**Figure 6:**
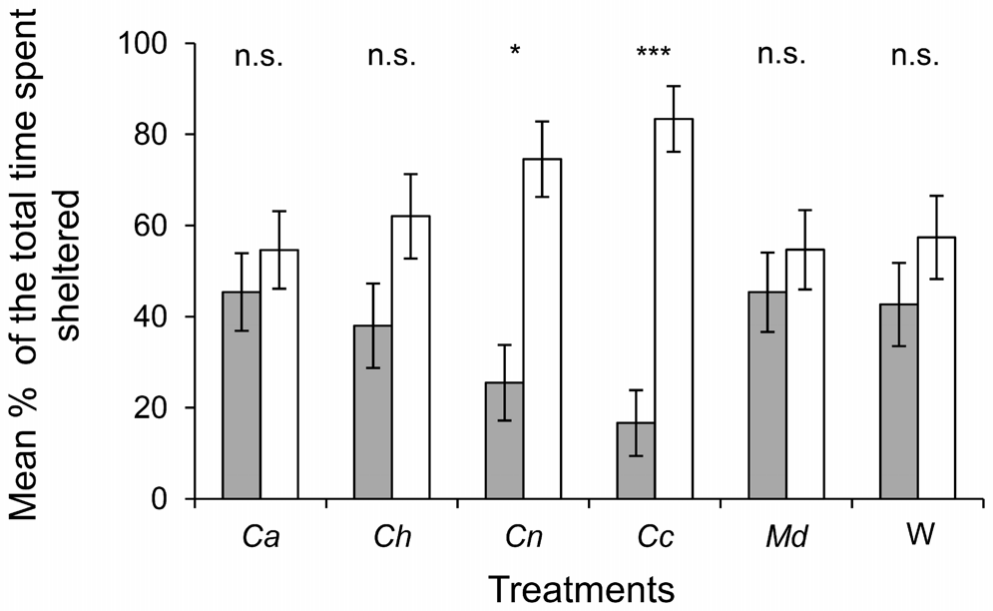
Proportion of time spent sheltered in either the treated or the control shelter for each of the 5 chemical treatment tested and the blank. Treatment applied in the treated shelter (dark boxes) for each test arena is indicated below each histogram pair: *Ca* (*Carabus auratus*), *Ch* (*Carabus hispanus*), *Cn* (*Carabus nemoralis*), *Cc* (*Carabus coriaceus*), *Md* (*Musca domestica*), W (strip of Whatman paper alone). Empty bars are the control (pure ethanol). Asterisks indicate significant differences: *P<0.05, ***P<0.001, n.s. not significant (Wilcoxon matched-pairs signed-ranks test). Bars = ±1 SE, n = 24.

**Figure 7:**
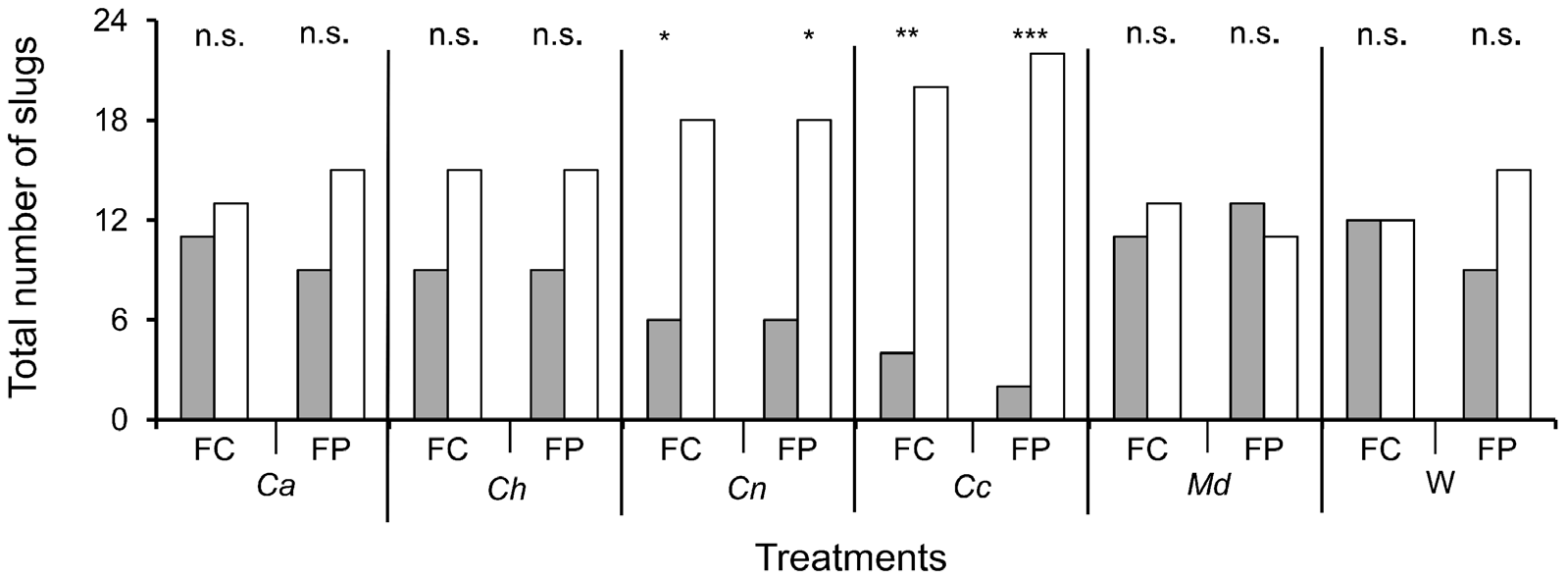
Total number of slugs having chosen a shelter with either treatment or control at first choice (FC) and total number of slugs found at the end of the experiment in each shelter according to the treatments (final position or FP). Dark boxes are the treated shelters, with: *Ca* (*Carabus auratus*), *Ch* (*Carabus hispanus*); *Cn* (*Carabus nemoralis*); *Cc* (*Carabus coriaceus*); *Md* (*Musca domestica*); W (strip of Whatman paper alone). Empty bars are the control shelters (pure ethanol). Asterisks indicate significant differences: *P<0.05, **P<0.01, ***P<0.001, n.s. not significant (McNemar’s test). Data collected from twenty-four replicates for each condition.

The slugs spent most of the duration of the experiment sheltered, regardless of the treatment used in the tested arenas (figure 5), with an average of 86% of the total experiment time spent in a sheltered area and only 14% in the lit area. However, it is interesting to note that the time spent by slugs in each shelter varied according to treatment (figure 6). In most of the cases, slugs spent an equivalent time in the control shelter and in the treated shelter, except in two situations. Indeed, there were no significant differences between time spent in the control shelter versus the treated shelter when cuticular extracts of C. *auratus*, *C. hispanus*, *M. domestica* were present or when only a blank Whatman paper was present. However, slugs spent significantly more time in the control shelter than in the shelter where cuticular extracts from either *C. nemoralis* or *C. coriaceus* were present (P = 0.015 and P<0.001 respectively, Wilcoxon matched-pairs signed-ranks test). The same observation was made regarding the slugs’ initial choice of shelter location (control vs. treatment shelter), along with their final choice of shelter location observed at the end of the test (figure 7). Indeed, when given a choice between the alcohol treatment and a shelter where cuticular extracts from *C. auratus*, *C. hispanus*, or *M. domestica* or a blank Whatman paper was present, the total number of slugs choosing the test shelter and control shelter were similar, as was the total number of slugs found present at the end of the replicates in the control shelter or the shelters that were treated with the previously cited treatments. By contrast, slugs significantly chose the control shelter as their first choice and were significantly more likely to be found in the control shelter at the end of the replicates, when cuticular extracts from *C. nemoralis* or *C. Coriaceus* were present in the test shelter. This effect was stronger when slugs were faced with *C. coriaceus* cuticular extracts than when faced with *C. nemoralis* (P = 0.001 and P = 0.014 respectively at first choice and P<0.001 and P = 0.014 respectively for the final position).

One-way ANOVA on transformed data (table 1) only showed significant differences between treatments regarding the time spent in the treated shelter (when expressed in seconds), and in the percentage of time spent in the treated shelter over the total trial duration. Tukey post-hoc multiple comparisons of observed means pointed out that, in both cases, the differences were observed between the cuticular extracts from *Carabus coriaceus* and *Musca domestica*.

**Table 1:** Comparison of eleven behavior parameters of *D. Reticulatum* measured for each treatment^a^.

	Treatments							
Behavior	*Ca*	*Ch*	*Cn*	*Cc*	*Md*	W	*F*_5,143_	*P*
Access latency to the first refuge (sec)	302.88	271.71	278.83	409.33	189.81	267.33	1.74	0.1302
Number of outings	0.542	0.583	0.667	0.625	0.875	0.917	0.6	0.697
Number of shelter permutations	0.417	0.417	0.333	0.208	0.458	0.542	0.62	0.685
Total time spent sheltered (sec)	3076.458	3239	3149.92	3010.63	3260.54	3002.92	1.8	0.1096
Total time spent sheltered (%)	85.457	89.97	87.5	83.63	90.57	83.41	1.97	0.0866
Time spent in the treated shelter (sec)	1345.375 ab	1202.54 ab	812.71 ab	438.5 b	1487.17 a	1339.17 ab	2.82	0.0187
Time spent in the control shelter (sec)	1731.083	2036.458	2337.208	2572.125	1773.375	1663.75	1.13	0.3451
Time spent in the treated shelter uponthe total trial duration (%)	37.372 ab	33.404 ab	22.575 ab	12.181 b	41.31 a	37.199 ab	2.47	0.0354
Time spent in the control shelter uponthe total trial duration (%)	48.086	56.568	64.922	71.448	49.26	46.215	1.52	0.188
Time spent in the treated shelter uponthe total time spent sheltered (%)	45.388	37.991	25.468	16.638	45.334	42.636	2.05	0.0749
Time spent in the control shelter uponthe total time spent sheltered (%)	54.612	62.009	74.532	83.362	54.666	57.364	2.05	0.0749

a Different letters indicate significant differences between treatments based on a Tukey post-hoc comparison of means test. *F* ratios are based on one-way ANOVAs. N = 24/treatment.

## Discussion

This study provides a reliable experimental pattern to conduct choice tests with slugs, which could be applied in any exploratory choice experiment dealing with the assessment of attractant or repulsive chemical cues in slugs.

The device, simple in its design, benefits from the natural proclivity displayed by slugs for dark places and from the functioning of their innate clock. Indeed, slug activity is the result of a complex interaction between an endogenous rhythm controlled by circadian rhythms, which is itself modulated by environmental conditions [73], [79], [83], [84]. Among these environmental factors, variations in temperature and light have a prominent influence on slug activity. For this reason, the beginning of the replicates was synchronized to the photoperiod cycle used in the slugs’ rearing room. Replicates thus began at the start of the photophase, while other abiotic factors (i.e. temperatures and relative humidity) were kept constant. This was done in order to balance the need for suitable levels of humidity and appropriate temperatures for slug activity (i.e. crawling), while using light as an external constraint to encourage slugs to find refuge and faithfully reproduce conditions found at dawn in the wild.

Slugs usually leave shelters after dusk and return to them around dawn with the first appearance of light. Incidentally, trapping slugs with artificial shelters that are then examined early the following morning is a common methodology employed for assessing slug populations in exposed fields [85]. *D. reticulatum*, like many other slugs, is well-known for making extensive use of shelters, especially during its inactive phase which typically occurs during daylight hours [76]. Because slugs have a soft body and depend on moist environments for their water demands, the use of refuges is thought to be a means of protection against the harsh external abiotic factors that threaten their bodily integrity [86]. Shelter seeking can thus be regarded as a self-maintenance behavior.

In the present study, the onset of activity in the slugs may also have been influenced by factors other than light. The handling required in order to place them properly in the center of the arenas was without a doubt the very first stimulus to trigger activity. Previous studies demonstrate that a burst of activity occurs in slugs that have just been handled [79]. However, it would have been difficult to allow the slugs to become acclimated to the arena undisturbed and still avoid head orientation bias.

Temperature also has a major influence on slug activity. Thus a noticeable increase in temperature in the lit area (from 15°C to 23°C for instance) due to an increase in light intensity, with shelters remaining cooler, should further encourage shelter seeking behavior in tested slugs. Indeed, 21°C has been identified as the threshold temperature above which slugs dramatically reduce their activity (conversely, their activity is triggered when temperatures fall below 21°C) [79], [83], [87] and tend to seek shelter [83]. However, the additional measures required to account for these influences could prove quite tedious and are unlikely to significantly improve results when compared to the present model, which already successfully fulfills all of the assessment criteria (cf. fig. 2, 3, 4 and 5).

The implementation of the device provided the means to demonstrate that cuticular extracts from two ground beetles, *C. coriaceus* and *C. nemoralis*, were effective in dissuading tested slugs from entering shelters where either of these stimuli were present. Armsworth *et al.* (2005) [36] showed that slugs display anti-predator behaviors in the presence of paper previously exposed to the crawling of *P. melanarius*. It also appears, based on a similar experiment, that these carabid beetles avoid paper that has been exposed to congeners and preferentially accumulate on unexposed control paper [88]. It is quite probable that these residual chemicals were left by the carabid beetles’ footprints.There are numerous studies reporting intra- and interspecific interactions mediated by insect footprints, which could serve to optimize foraging activities in bumble bees [89]–[92] or act as host location kairomones [93], [94]. In many cases, the origins of these scent markers remain unclear, as they could be actively (i.e. glandular origin) or passively secreted (merely residual footprints). In the latter case, it is likely that cuticular hydrocarbons (CHCs) are involved, as is suspected to be the case with *Spodoptera frugiperda* caterpillar footprint compounds, which betray the presence of the caterpillar to the benefit of the Braconidae parasitoid, *Cotesia marginiventris*.

CHCs are present throughout the outer surface of the insects’ cuticle and have long been recognized for their many diverse functions in insects, including both physiological and ecological roles (see [95]–[97]). Cuticular extracts are generally obtained using dichloromethane, pentane, chloroform or hexane. These solvents, being strong nonpolars (but in a lesser extent, that being said, regarding dichloromethane), are effective to extract surface cuticular lipids. However these have been known to contaminate the cuticular fraction with unrelated and undesired materials (internal glands, lipid extracts, etc.) [95], [98]. Ethanol, in contrast, is a polar molecule that allows affinity for a wider spectrum of molecules species, to both hydrophobic and hydrophilic molecules and it was more suitable for our experiment, because of its nontoxicity towards slugs in the quantity used. Ethanol has been reported to conserve body extract hallmarks for at least one month after preparation [78], [99]. It can thus be hypothesized that slugs were deterred by light chemical compounds which were leached out of the outer surface of the ground beetle’s exoskeleton, which is typically deposited on the ground during regular activity.

*C. coriaceus* is heliciphagous, capable of overcoming the secondary defenses of large snails on which they feed [100]. Since its body is not shaped to force its way through the snails’ aperture, like in *Cychrus spp.*, it appears to rely on the strength of its jaws to break the shell [101]. *C. coriaceus* also feeds on slugs and, given the previous considerations, it is likely that slugs’ mucus exudation is ineffective in deterring attacks from this predatory beetle. *C. coriaceus* are eurytopic beetles that can be found in forests but demonstrate a preference for ecotones at forest edges and may even be found in open habitats, such as parks and gardens [102], [103], a preference related to eye structure [103]. In this study, *C. coriaceus* were caught in hedges adjacent to fields where *D. reticulatum* were numerous. These two species are sympatric in west and central Europe and thus *D. reticulatum* may account for a significant part of the diet of *C. coriaceus*, sufficient at least to represent such a major threat that the innate ability to detect this predator is mandatory for the survival of young slugs.

Likewise, *C. nemoralis* is found in similar habitats to *C. coriaceus*. It is generally encountered in open, anthropogenically altered areas like gardens and parks [51], [104], or near set-aside arable areas [105], [106]. Observations of diet preferences for *C. nemoralis* are somewhat conflicting, some consider it to be a specialist [107] while others view it as a generalist [108], [109]. However, several studies have reported its skill in preying upon mollusks, notably on slugs at both a physiological level and in laboratory experiments. In particular, Digweed 1994 demonstrated that *C. nemoralis* orientate to *D. reticulatum* mucus trails and are able to consume large *A. lusitanicus*, up to 1.3 g, with a preference for slugs weighing less than 1 g [51]. Despite mucus from arionids being stickier than *D. reticulatum,* no marked consumption preference for either *A. lusitanicus* or *D. reticulatum* was noticed. *C. nemoralis* proved to be an efficient slug-killer, targeting its attack to the posterior end of the slugs, and often resulting in prey death at first strike [51], [107]. Ayre (1995) [107] conducted a comparison consumption test on *D. reticulatum* weighing between 0.1 to 0.7 g using a mix of generalist and specialist ground beetles, and *C. nemoralis* appeared as efficient as the specialized *C. caraboides* and *C. violaceus* ground beetles in feeding on slugs. These observations indicate that this beetle may be capable of handling even large *D. reticulatum*. Thus, small slugs, like those used in the experiments, would have limited means of self-defense against *C. nemoralis*. Thus, the ability to detect the presence of this ground beetle may be a selective advantage, allowing to the slug to anticipate and avoid potentially risky areas.

For prey, avoiding predation threat has a cost, because it results in a shift of trade-offs [2]. Detection of predators is thus selective, as responding to cues from non-hostile organisms can be detrimental to prey, causing them to lose opportunities to perform activities that are essential to fitness. This was consistent with study results, as the slugs did not avoid the shelter treated with *M. domestica* extracts. Being a neutral insect, the house flies do not represent a threat to slugs. The two other cuticular extracts from ground beetles, *C. auratus* and *C. hispanus*, were also ineffective in deterring slugs from entering the treated shelters. It is also possible that the innate ability to perceive predators displayed by juvenile slugs is designed to act solely against the most relevant chemical cues, which correspond to their most threatening predators.

Regarding *C. hispanus,* explanations may suffer from lack of data, which is perhaps due to its endemic status in a narrow area of France. Nevertheless, from an ecological standpoint, some major distinctions between *C. hispanus*, *C. coriaceus* and *C. nemoralis* can be drawn. Indeed, *C. hispanus* is primarily a forest species with good tree climbing skills and an oligophagous diet [110] ranging from snails to fruits, which may account for its reduced threat to slugs.

Results obtained with *C. auratus* are somewhat surprising. Despite it being a highly voracious species and a well-known friend to gardeners, the cuticular extracts did not have a crucial impact on slug behavior. In fact, this species shares several features with the previous species. For example, *C. auratus* prefers open habitats, like *C. coriaceus* and *C. nemoralis*, whilst also being found in forests. The species is also very opportunistic, able to climb trees for hunting, as *C. hispanus* does. Thus, it is possible that *C. auratus*, like *C. hispanus*, prey on slugs to a lesser extent than *C. coriaceus* and *C. nemoralis*.

Studies by Armsworth *et al.* (2005) [36] show that slugs display anti-predator behaviors (area avoidance, increased speed, reduced turning rate) in the presence of papers previously exposed to the crawling of *P. melanarius*, a generalist species. In their experiments, slugs were caught in the wild making it impossible to account for life history traits. Thus the possibility that they learned to recognize *P. melanarius* through experience cannot be excluded, as it is a well-documented feature for a variety of taxa in many studies [3], [111]–[113]. On the contrary, the slugs employed in our study were predator-naive, so they may have failed to respond to *C. auratus* and *C. hispanus* chemical cues because the two species may be less serious predators for slugs, and their cues may require learning.

In addition, prey often respond more readily to chemical cues of a predator fed with conspecifics or, at least, with a closely related species. For instance, Persons *et al.* (2001) [61] showed that anti-predatory behaviors against the wolf spider *Pardosa milvina* were stronger in the presence of cues from the predatory wolf spider *Hogna helluo* fed *P. milvina* than when fed the crickets *Acheta domesticus*. Likewise, Lefcort *et al.* (2006) [35] demonstrated that *Theba pisana* modulates its response according to the diet of *Carabus carabus*: anti-predator behaviors are more obvious when *T. pisana* is presented with feces cues from snail-fed ground beetles than in the presence of feces cues from *C. carabus* fed with chicken. In this study, the ground beetles are fed with *X. derbentina* which means that tested slugs do not react to conspecific dead material, like apneumones [114]. *X. derbentina* is a xerothermophilic land snail species native to Eastern Mediterranean Europe, accidently introduced in southeast France during the 1940s [115], [116] and now well-established in Provence [117]. *X. derbentina* and *D. reticulatum*, while both belonging to the Stylommatophora order, are not only fairly phylogenetically distant from each other, but occur in different ecological habitats and thus experience very different constraints. Nevertheless, the juvenile slugs showed strong avoidance to chemical cues from *C. nemoralis* and *C. coriaceus.* This avoidance was quickly performed, sustained and irreversible for the duration of the experiment (see fig. 6 and 7), indicating that these ground beetles must exert such a selection pressure on slugs that even the consumption of a distant terrestrial gastropod cannot alter the young *D. reticulatum’s* perception of these beetles as a foe. In contrast, the absence of avoidance in the presence of cuticular extracts from *C. auratus* or *C. hispanus* does not necessarily imply that they are inherently non-hazardous predators for slugs. Additional suitable cues, in the form of diet-based by-products (i.e., a diet based on related slugs species), may be required for the naive slugs to perceive these ground beetles as an actual threat. It is also possible that the cue was not eluted by ethanol, but it could be present in other solvents with greater affinity for hydrophobic compounds than ethanol, such as dichloromethane or hexane. Moreover, it cannot be excluded that the cue is concentration-dependant. From this point of view, a correlation can be drawn between the two ground beetle cuticular extracts that did not affect the slugs’ behavior and their age. Indeed, *C. hispanus* and *C.auratus* cuticular extracts were made about 6 and 4 months respectively before the beginning of the trials, compared with the 3 months of standby for the other cuticular extracts. We can thus not rule out that the oldest solutions were somewhat altered in such extent that they could no more operate on slugs.

Refuges can be useful to prey devoid of efficient secondary defenses. Turner (1996) [118] found that in the presence of the specialist feeder fish, *Lepomis gibbosus*, *Physella* move under covered habitats to reduce the risk of being caught. Similarly, Symondson (1993) [119] deduced from his results that *D. reticulatum* uses large lettuce leaves as a means of protection against the generalist carabid beetle *A. parallelepipedus* by blocking its access. The results of this study are the first to expose the impact of chemical residuals from two specialist ground beetles in influencing the shelter choices of slugs. The slugs may be forced to travel further to find a suitable refuge, which could have a detrimental impact on fitness. As their movements involve the excretion of large amounts of mucus, traveling a greater distance may affect their body water-content [120] and increase their vulnerability through prolonged exposure to adverse abiotic factors (increased risk of desiccation) and/or to predators. Questions may be raised as to the extent of the effect of ground beetle chemical cues on other important self-maintenance behaviors, such as foraging. Further experiments may be conducted to explore whether slugs are able to refine predation risk assessment according to predator diet, or whether they can learn to recognize predators based on diet. These investigations could be performed either with ethanol/dichloromethane cuticular extracts from ground beetles, or using residuals from the crawling of live predators left on substratum (such as Whatman paper). Finally, chemical analysis must be carried out so as to reveal the nature of the chemical compounds that mediate this predator-prey interaction before being put to the test through experiments on slugs’ behavior.

## References

[1] Kats LB, Dill LM (1998) The scent of death: chemosensory assessment of predation risk by prey animals. Ecoscience 5: 361–394.

[2] Lima SL, Dill LM (1990) Behavioral decisions made under the risk of predation: a review and prospectus. Can J Zool 68: 619–640 10.1139/z90-092

[3] Dicke M, Grostal P (2001) Chemical Detection of Natural Enemies by Arthropods: An Ecological Perspective. Annu Rev Ecol Syst 32: 1–23 10.1146/annurev.ecolsys.32.081501.113951

[4] Vermeij GJ (1994) The Evolutionary Interaction Among Species: Selection, Escalation, and Coevolution. Annu Rev Ecol Syst 25: 219–236 10.1146/annurev.es.25.110194.001251

[5] Vermeij GJ (1982) Unsuccessful Predation and Evolution. Am Nat 120: 701–720.

[6] Sih A (1987) Predators and prey life styles: an evolutionary and ecological overview. In: Predation: Direct and Indirect Impacts on Aquatic Communities. Kerfoot WC, Sih A, editors Hanover: Hanover, New Hampshire: University Press of New England.

[7] Edmunds M (1974) Defence in animal: a survey of anti-predator defences. Edmunds M, editor Longman, Harlow, England.

[8] Chivers DP, Smith RJF (1998) Chemical alarm signalling in aquatic predator-prey systems: A review and prospectus. Ecoscience 5: 338–352.

[9] Ferrari MCO, Sih A, Chivers DP (2009) The paradox of risk allocation: a review and prospectus. Anim Behav 78: 579–585 10.1016/j.anbehav.2009.05.034

[10] Wisenden BD (2000) Olfactory assessment of predation risk in the aquatic environment. Philos Trans R Soc Lond B Biol Sci 355: 1205–1208 10.1098/rstb.2000.0668 11079399 PMC1692838

[11] Wisenden BD, Millard MC (2001) Aquatic flatworms use chemical cues from injured conspecifics to assess predation risk and to associate risk with novel cues. Anim Behav 62: 761–766 10.1006/anbe.2001.1797

[12] Hara TJ (1992) Overview and introduction. In: Hara TJ, editor. Fish Chemoreception. New-York. 1–12.

[13] Storm JJ, Lima SL (2008) Predator-naïve fall field crickets respond to the chemical cues of wolf spiders. Can J Zool 86: 1259–1263 10.1139/Z08-114

[14] Slifer EH (1970) The structure of arthropod chemoreceptors. Annu Rev Entomol 15: 121–142 10.1146/annurev.en.15.010170.001005

[15] Chapman RF (1998) Chemoreception. In: Chapman RF, editor. The Insects - Structure and Function. Cambridge: Cambridge University Press. 636–654.

[16] Zacharuk RY (1985) Antannae and sensilla. In: Kerkut GA, Gilbert LJ, editors. Comprehensive Insect Physiology, Biochemistry and Pharmacology. Oxford: Oxford: Pergamon Press. 1–69.

[17] Zacharuk RY (1980) Ultrastructure and Function of Insect Chemosensilla. Annu Rev Entomol 25: 27–47 10.1146/annurev.en.25.010180.000331

[18] Dalesman S, Rundle SD, Cotton PA (2007) Predator regime influences innate anti-predator behaviour in the freshwater gastropod *Lymnaea stagnalis* . Freshw Biol 52: 2134–2140 10.1111/j.1365-2427.2007.01843.x

[19] Orr MV, El-Bekai M, Lui M, Watson K, Lukowiak K (2007) Predator detection in Lymnaea stagnalis. J Exp Biol 210: 4150–4158 10.1242/jeb.010173 18025014

[20] Turner AM, Turner SE, Lappi HM (2006) Learning, memory and predator avoidance by freshwater snails: effects of experience on predator recognition and defensive strategy. Anim Behav 72: 1443–1450 10.1016/j.anbehav.2006.05.010

[21] Ferrari MCO, Wisenden BD, Chivers DP (2010) Chemical ecology of predator-prey interactions in aquatic ecosystems: a review and prospectus. Can J Zool 88: 698–724 10.1139/Z10-029

[22] Jacobsen HP, Stabell OB (2004) Antipredator behaviour mediated by chemical cues: the role of conspecific alarm signalling and predator labelling in the avoidance response of a marine gastropod. Oikos 104: 43–50 10.1111/j.0030-1299.2004.12369.x

[23] Croll RP (1983) Gastropod chemoreception. Biol Rev 58: 293–319 10.1111/j.1469-185X.1983.tb00391.x

[24] Zaitseva OV (1994) Structural Organization of the Sensory Systems of the Snail. Neurosci Behav Physiol 24: 47–57 10.1007/BF02355652 8208381

[25] Zaitseva OV (1999) Principles of the structural organization of the chemosensory systems of freshwater gastropod mollusks. Neurosci Behav Physiol 29: 581–593 10.1007/BF02461151 10596795

[26] Kohn AJ (1961) Chemoreception in gastropod molluscs. Am Zool 1: 291–308.

[27] Barker GM (2001) The Biology of Terrestrial Molluscs. Barker GM, editor Hamilton: CABI Publishing.

[28] South A (1992) Terrestrial Slugs: Biology, Ecology, Control. London, New-York: Chapman & Hall.

[29] Chase R (1986) Lessons from snail tentacles. Chem Senses 11: 411–426 10.1093/chemse/11.4.411

[30] Zieger MV, Vakoliuk IA, Tuchina OP, Zhukov VV, Meyer-Rochow VB (2009) Eyes and vision in Arion rufus and Deroceras agreste (Mollusca; Gastropoda; Pulmonata): What role does photoreception play in the orientation of these terrestrial slugs? Acta Zool 90: 189–204 10.1111/j.1463-6395.2008.00369.x

[31] Hamilton PV, Winter MA (1984) Behavioural responses to visual stimuli by the snails Tectarius muricatus, Turbo castanea, and Helix aspersa. Anim Behav 32: 51–57 10.1016/S0003-3472(84)80323-1

[32] Eakin RM, Brandenburger JL (1975) Understanding a snail’s eye at a snail’s pace. Am Zool 15: 851–863 10.1093/icb/15.4.851

[33] Barker GM (2004) Natural enemies of terrestrial gastropods. Barker GM, editor Hamilton: CABI Publishing.

[34] Symondson WOC (2004) Coleoptera (Carabidae, Staphylinidae, Lampyridae, Drilidae and Silphidae) as predators of terrestrial gastropods. In: Barker GM, editor. Natural enemies of terrestrial molluscs. Hamilton: CABI. 37–84.

[35] Lefcort H, Ben-Ami F, Heller J (2006) Terrestrial snails use predator-diet to assess danger. J Ethol 24: 97–102 10.1007/s10164-005-0168-0

[36] Armsworth CG, Bohan DA, Powers SJ, Glen DM, Symondson WOC (2005) Behavioural responses by slugs to chemicals from a generalist predator. Anim Behav 69: 805–811 10.1016/j.anbehav.2004.07.009

[37] Symondson WOC (1989) Biological control of slugs by carabids. In: Henderson IF, editor. Slugs and snails in world agriculture. Thornton Heath: British Crop Protection Council. 295–300.

[38] Bohan DA, Bohan AC, Glen DM, Symondson WOC, Wiltshire CW, et al (2000) Spatial dynamics of predation by carabid beetles on slugs. J Anim Ecol 69: 367–379 10.1046/j.1365-2656.2000.00399.x

[39] McKemey AR, Symondson WOC, Glen DM, Brain P (2001) Effects of slug size on predation by Pterostichus melanarius (Coleoptera: Carabidae). Biocontrol Sci Technol 11: 81–91 10.1080/09583150020029763

[40] Symondson WOC, Sunderland KD, Greenstone MH (2002) Can generalist predators be effective biocontrol agents? Annu Rev Entomol 47: 561–594 10.1146/annurev.ento.47.091201.145240 11729085

[41] Symondson WOC (1994) The potential of Abax parallelepipedus (Col.: Carabidae) for mass breeding as a biological control agent against slugs. Entomophaga 39: 323–333 10.1007/BF02373037

[42] Asteraki EJ (1993) The potential of carabid beetles to control slugs in grass/clover swards. Entomophaga 38: 193–198 10.1007/BF02372553

[43] Kromp B (1999) Carabid beetles in sustainable agriculture: a review on pest control efficacy, cultivation impacts and enhancement. Agric Ecosyst Environ 74: 187–228 10.1016/S0167-8809(99)00037-7

[44] Martin TJ, Kelly JR (1986) The effects of changing agriculture on slugs as pests of cereals. Proceedings 1986 Brighton Crop Protection Conference - Pests and Diseases. Farnham: BCPC. 441–424.

[45] Bourne NB, Jones GW, Bowen ID (1988) Slug feeding behaviour in relation to control with molluscicidal baits. J Molluscan Stud 54: 327–338 10.1093/mollus/54.3.327

[46] Bailey SER, Wedgwood MA (1991) Complementary video and acoustic recordings of foraging by two pest species of slugs on non-toxic and molluscicidal baits. Ann Appl Biol 119: 163–176 10.1111/j.1744-7348.1991.tb04855.x

[47] Homeida AM, Cooke RG (1982) Pharmacological aspects of metaldehyde poisoning in mice. J Vet Pharmacol Ther 5: 77–81 10.1111/j.1365-2885.1982.tb00500.x 6178838

[48] Johnson IP, Flowerdew JR, Hare R (1992) Populations and diet of small rodents and shrews in relation to pesticide usage. In: Greig-Smith PW, Frampton G, Hardy T, editors. Pesticides, cereal farming and the environment: the Boxworth project. 144–156.

[49] Corfield GS, Connor LM, Swindells KL, Johnson VS, Raisis AL (2008) Intussusception following methiocarb toxicity in three dogs. J Vet Emerg Crit Care 18: 68–74 10.1111/j.1476-4431.2007.00271.x

[50] Ayre K (2001) Effect of predator size and temperature on the predation of *Deroceras reticulatum* (Müller) (Mollusca) by carabid beetles. J Appl Entomol 125: 389–395 10.1046/j.1439-0418.2001.00568.x

[51] Hatteland BA, Grutle K, Mong CE, Skartveit J, Symondson WOC, et al (2010) Predation by beetles (Carabidae, Staphylinidae) on eggs and juveniles of the Iberian slug *Arion lusitanicus* in the laboratory. Bull Entomol Res 100: 559–567 10.1017/S0007485309990629 20158927

[52] Kielty JP, Allen-Williams LJ, Underwood N, Eastwood EA (1996) Behavioral responses of three species of ground beetle (Coleoptera: Carabidae) to olfactory cues associated with prey and habitat. J Insect Behav 9: 237–250 10.1007/BF02213868

[53] Oberholzer F, Frank T (2003) Predation by the Carabid Beetles *Pterostichus melanarius* and *Poecilus cupreus* on Slugs and Slug Eggs. Biocontrol Sci Technol 13: 99–110 10.1080/0958315021000054421

[54] Symondson WOC, Glen DM, Wiltshire CW, Langdon CJ, Liddekk JE (1996) Effects of Cultivation Techniques and Methods of Straw Disposal on Predation by *Pterostichus melanarius* (Coleoptera: Carabidae) Upon Slugs (Gastropoda: Pulmonata) in an Arable Field. J Appl Ecol 33: 741–753.

[55] Symondson WOC, Glen DM, Ives AR, Langdon CJ, Wiltshire CW (2002) Dynamics of the relationship between a generalist predator and slugs over five years. Ecology 83: 137–147 10.1890/00129658(2002)0830137:DOTRBA2.0.CO2

[56] Mair J, Port GR (2002) The influence of mucus production by the Slug, Deroceras Reticulatum, on predation by Pterostichus madidus and Nebria brevicollis (Coleoptera: Carabidae). Biocontrol Sci Technol 12: 325–335 10.1080/09583150220128112

[57] Mair J, Port GR (2001) Predation by the carabid beetle *Pterostichus madidus* and *Nebria brevicollis* is affected by size and condition of the prey slug *Deroceras reticulatum* . Agric For Entomol 3: 99–106 10.1046/j.1461-9563.2001.00093.x

[58] Pakarinen E (1994) The Importance of Mucus As a Defence Against Carabid Beetles By the Slugs *Arion Fasciatus* and *Deroceras Reticulatum* . J Molluscan Stud 60: 149–155 10.1093/mollus/60.2.149

[59] Pakarinen E (1994) Autotomy in Arionid and Limacid Slugs. J Molluscan Stud 60: 19–23 10.1093/mollus/60.1.19

[60] Deyrup-Olsen I, Martin AW, Paine RT (1986) The autotomy escape response of the terrestrial slug *Prophysaon foliatum* (Pulmonata: Arionidae). Malacologia 27: 307–311.

[61] Persons MH, Walker SE, Rypstra AL, Marshall SD (2001) Wolf spider predator avoidance tactics and survival in the presence of diet-associated predator cues (Araneae: Lycosidae). Anim Behav 61: 43–51 10.1006/anbe.2000.1594 11170695

[62] Barnes MC, Persons MH, Rypstra AL (2002) The effect of predator chemical cue age on antipredator behavior in the wolf spider Pardosa milvina (Araneae: Lycosidae). J Insect Behav 15: 269–281 10.1023/A:1015493118836

[63] Bell RD, Rypstra AL, Persons MH (2006) The Effect of Predator Hunger on Chemically Mediated Antipredator Responses and Survival in the Wolf Spider *Pardosa milvina* (Araneae: Lycosidae). Ethology 112: 903–910 10.1111/j.1439-0310.2006.01244.x

[64] Persons MH, Rypstra AL (2001) Wolf spiders show graded antipredator behavior in the presence of chemical cues from different sized predators. J Chem Ecol 27: 2493–2504 10.1023/A:1013679532070 11789954

[65] Lehmann LM, Walker SE, Persons MH (2004) The Influence of Predator Sex on Chemically Mediated Antipredator Response in the Wolf Spider Pardosa milvina (Araneae: Lycosidae). Ethology 110: 323–339 10.1111/j.1439-0310.2004.00972.x

[66] Kiesecker JM, Chivers DP, Blaustein AR (1996) The use of chemical cues in predator recognition by western toad tadpoles. Anim Behav 52: 1237–1245 10.1006/anbe.1996.0271

[67] Puttlitz MH, Chivers DP, Kiesecker JM, Blaustein AR (1999) Threat-sensitive Predator Avoidance by Larval Pacific Treefrogs (Amphibia, Hylidae). Ethology 105: 449–456 10.1046/j.1439-0310.1999.00416.x

[68] Wahle RA (1992) Body-Size Dependent Anti-Predator Mechanisms of the American Lobster. Oikos 65: 52–60.

[69] Ferrari MCO, Trowell JJ, Brown GE, Chivers DP (2005) The role of learning in the development of threat-sensitive predator avoidance by fathead minnows. Anim Behav 70: 777–784 10.1016/j.anbehav.2005.01.009

[70] Ferrari MCO, Gonzalo A, Messier F, Chivers DP (2007) Generalization of learned predator recognition: an experimental test and framework for future studies. Proc R Soc B Biol Sci 274: 1853–1859 10.1098/rspb.2007.0297 PMC2270927 17519190

[71] Newell PF (1966) The nocturnal behaviour of slugs. Med Biol Illus 16: 146–159. 5943726

[72] Barker GM (2002) Molluscs as Crop Pests. Thomas T &. Barker GM, editor Hamilton: CABI Publishing.

[73] Wareing DR, Bailey SER (1985) The effects of steady and cycling temperatures on the activity of the slug *Deroceras reticulatum* . J Molluscan Stud 51: 257–266.

[74] Lewis RD (1969) Studies on the locomotor activity of the slug *Arion ater* (Linnaeus) I. Humidity, Temperatur and Light reactions. Malacologia 7: 295–306.

[75] Newell PF (1968) The measurement of light and temperature as factors controlling the surface activity of the slug *Agriolimax reticulatus* (Müller). In: Wadsworth RM, editor. The measurement of environmental factors in terrestrial ecology. Oxford: Blackwell. 141–146.

[76] Hommay G, Lorvelec O, Jacky F (1998) Daily activity rhythm and use of shelter in the slugs *Deroceras reticulation* and *Arion distinctus* under laboratory conditions. Ann Appl Biol 132: 167–185 10.1111/j.1744-7348.1998.tb05193.x

[77] Rollo CD, Borden JH, Casey IB (1995) Endogenously Produced Repellent from American Cockroach (Blattaria: Blattidae): Fuction in Death Recognition. Environ Entomol 24: 116–124.

[78] Rollo CD, Czvzewska E, Borden JH (1994) Fatty acid necromones for cockroaches. Naturwissenschaften 81: 409–410 10.1007/BF01132695

[79] Dainton BH (1954) The activity of slugs. I. The induction of activity by changing temperatures. J Exp Biol 31: 165–187.

[80] Chase R (1982) The Olfactory Sensitivity of Snails, Achatina fulica. J Comp Physiol 148: 225–235.

[81] Underwood AJ (1981) Techniques of analysis of variance in experimental marine biology and ecology. Oceanogr Mar Biol an Annu Rev 19: 513–605.

[82] Krebs CJ (1989) Ecological Methodology. New-York: Harper & Row.

[83] Rollo CD (1991) Endogenous and exogneous regulation of activity in *Deroceras reticulatum*, a wheather-sensitive terrestrial slug. Malacologia 33: 199–220.

[84] Dainton BH (1954) The activity of slugs. II. The effects of light and air currents. J Exp Biol 31: 188–197.

[85] Hommay G, Kienlen JC, Jacky F, Gertz C (2003) Daily variation in the number of slugs under refuge traps. Ann Appl Biol 142: 333–339 10.1111/j.1744-7348.2003.tb00258.x

[86] Grewal PS, Grewal SK, Taylor RAJ, Hammond RB (2001) Application of Molluscicidal Nematodes to Slug Shelters: A Novel Approach to Economic Biological Control of Slugs. Biol Control 22: 72–80 10.1006/bcon.2001.0958

[87] Dainton BH, Wright J (1985) Falling Temperature Stimulates Activity in the Slug Arion Ater. J Exp Biol 118: 439–443.

[88] Guy AG, Bohan DA, Powers SJ, Reynolds AM (2008) Avoidance of conspecific odour by carabid beetles: a mechanism for the emergence of scale-free searching patterns. Anim Behav 76: 585–591 10.1016/j.anbehav.2008.04.004

[89] Stout JC, Goulson D, Allen JA (1998) Repellent scent-marking of flowers by a guild of foraging bumblebees (Bombus spp.). Behav Ecol Sociobiol 43: 317–326 10.1007/s002650050497

[90] Schmitt U, Bertsch A (1990) Do foraging bumblebees scent-mark food sources and does it matter? Oecologia 82: 137–144 10.1007/BF00318545 28313149

[91] Gawleta N, Zimmermann Y, Eltz T (2005) Repellent foraging scent recognition across bee families. Apidologie 36: 325–330 10.1051/apido:2005018

[92] Witjes S, Eltz T (2009) Hydrocarbon footprints as a record of bumblebee flower visitation. J Chem Ecol 35: 1320–1325 10.1007/s10886-009-9720-7 20013038

[93] Rostás M, Wölfling M (2009) Caterpillar footprints as host location kairomones for Cotesia marginiventris: persistence and chemical nature. J Chem Ecol 35: 20–27 10.1007/s10886-009-9590-z 19153795

[94] Wölfling M, Rostás M (2009) Parasitoids use chemical footprints to track down caterpillars. Commun Integr Biol 2: 353–355 10.1007/s10886-009-9590-z.These 19721889 PMC2734046

[95] Howard RW (1993) Cuticular hydrocarbons and Chemical communication. In: Stanley-Samuelson DW, Nelson DR, editors. Insect lipids: chemistry, biochemistry, and biology. Lincoln: Lincoln: University of Nebraska Press. 179–226.

[96] Howard RW, Blomquist GJ (2005) Ecological, behavioral, and biochemical aspects of insect hydrocarbons. Annu Rev Entomol 50: 371–393 10.1146/annurev.ento.50.071803.130359 15355247

[97] Chapman RF (1998) Excretion and salt and water regulation. In: Chapman RF, editor. The Insects - Structure and Function. Cambridge: Cambridge University Press. 478–508.

[98] Bagnères AG, Morgan ED (1990) A simple method for analysis of insect cuticular hydrocarbons. J Chem Ecol 16: 3263–3276 10.1007/BF00982097 24263428

[99] Yao M, Rosenfeld J, Attridge S, Sidhu S, Aksenov V, et al (2009) The Ancient Chemistry of Avoiding Risks of Predation and Disease. Evol Biol 36: 267–281 10.1007/s11692-009-9069-4

[100] Skłodowski JJW (2005) Interspecific body size differentiation in Carabus assemblages in the Białowieża Primeval Forest, Poland. In: Lövei GL, Toft S, editors. European Carabidology 2003. Proceedings of the 11th European Carabidologist Meeting. Foulum, Denmark: DIAS Reports no. 114, Vol. 114: 291–303.

[101] Sturani M (1962) Osservazioni e ricerche biologiche sul genere *Carabus* L.(sl).(Coleoptera, Carabidae). Mem della Soc Entomol Ital 41: 85–202.

[102] Riecken U, Raths U (1996) Use of radio telemetry for studying dispersal and habitat use of Carabus coriaceus L. Ann Zool Fennici. 33: 109–116.

[103] Talarico FF, Romeo M, Massolo A, Brandmayr P, Zetto T (2007) Morphometry and eye morphology in three species of *Carabus* (Coleoptera: Carabidae) in relation to habitat demands. J Zool Syst Evol Res 45: 33–38 10.1111/j.1439-0469.2006.00394.x

[104] Niemelä JK, Spence JR (1994) Distribution of Forest Dwelling Carabids (Coleoptera): Spatial Scale and the Concept of Communities. Ecography (Cop) 17: 166–175 10.1111/j.1600-0587.1994.tb00090.x

[105] Kennedy P (1994) The distribution and movement of ground beetles in relation to set-aside arable land. In: Desender K, Dufrene M, Loreau M,Cuff ML, Maelfait J-P, editors. Carabid Beetles: Ecology and Evolution. Dordecht: Kluwer Academic. 439–444.

[106] Gruttke H (1994) Dispersal of carabid species along a linear sequence of young hedge plantations. In: Desender K, Dufrêne M, Loreau M, Luff ML, Maelfait J-P, editors. Carabid Beetles: Ecology and Evolution. Dordrecht: Kluwer Academic Publishers. 299–303.

[107] Ayre K (1995) Evaluation of carabids as predators of slugs in arable land. Thesis, University of Newcastle Upon Tyne.

[108] Hengeveld R (1980) Qualitative and quantitative aspects of the food of ground beetles (Coleoptera, Carabidae): a review. Netherlands J Zool 30: 555–563 10.1163/002829679X00188

[109] Digweed SC (1994) Detection of mucus-producing prey by *Carabus nemoralis* Mueller and *Scaphinotus marginatus* Fischer (Coleoptera: Carabidae). Coleopt Bull 48: 361–369.

[110] Turin H, Penev L, Casale A, Arndt E, Assmann T, et al. (2003) Species accounts. In: Turin H, Penev L, Casale A, editors. The Genus Carabus in Europe. A synthesis. Leiden: Pensoft Publishers, Sofia-Moscow & European Invertebrate Survey. 141–286.

[111] Chivers DP, Wisenden BD, Smith JF (1996) Damselfly larvae learn to recognize predators from chemical cues in the predators diet. Anim Behav 52: 315 10.1006/anbe.1996.0177

[112] Ferrari MCO, Capitania-Kwok T, Chivers DP (2006) The role of learning in the acquisition of threat-sensitive responses to predator odours. Behav Ecol Sociobiol 60: 522–527 10.1007/s00265-006-0195-z

[113] Mirza RS, Ferrari MCO, Kiesecker JM, Chivers DP (2006) Responses of American toad tadpoles to predation cues: behavioural response thresholds, threat-sensitivity and acquired predation recognition. Behaviour 143: 877–889 10.1163/156853906778017926

[114] Nordlund DA, Lewis WJ (1976) Terminology of chemical releasing stimuli in intraspecific and interspecific interactions. J Chem Ecol 2: 211–220 10.1007/BF00987744

[115] Kiss L, Labaune C, Magnin F, Aubry S (2005) Plasticity of the Life Cycle of *Xeropicta Derbentina* (Krynicki, 1836), a Recently Introduced Snail in Mediterranean France. J Molluscan Stud 71: 221–231 10.1093/mollus/eyi030

[116] Regteren A Van (1960) On the occurrence of a species of *Xeropicta* in France. Basteria 24: 21–26.

[117] Labaune C, Magnin F (1999) Un escargot nouveau venu dans le Luberon et en Provence: *Xeropicta derbentina* (Krinicki, 1836). Courr Sci Du Parc Nat Régional Luberon 3: 102–110.

[118] Turner AM (1996) Freshwater snails alter habitat use in response to predation. Anim Behav 51: 747–756 10.1006/anbe.1996.0079

[119] Symondson WOC (1993) The Effects of Crop Development Upon Slug Distribution and Control by Abax Parallelepipedus (Coleoptera, Carabidae). Ann Appl Biol 123: 449–457 10.1111/j.1744-7348.1993.tb04107.x

[120] Lyth M (1982) Water-content of slugs (Gastropoda: Pulmonata) maintained in standardised culture conditions. J Molluscan Stud 48: 214–218.

