# Correction: Chemical Compounds Related to the Predation Risk Posed by Malacophagous Ground Beetles Alter Self-Maintenance Behavior of Naive Slugs (*Deroceras reticulatum*)

**DOI:** 10.1371/annotation/0b417407-f2e1-45ef-9337-8126b796f0f5

**Published:** 2013-12-10

**Authors:** Piotr Bursztyka, Dominique Saffray, Céline Lafont-Lecuelle, Antoine Brin, Patrick Pageat

Affiliation number 1 for the first and fourth authors is incorrect. The correct affiliation number 1 is: Biodiversité des Systèmes Agricoles et Naturels UMR INRA/INPT 1201 Dynafor, Engineering School of Purpan, Toulouse, Haute-Garonne, France. 

